# Automated chemical synthesis. Part 2: Interfacing strategies

**DOI:** 10.1155/S1463924683000267

**Published:** 1983

**Authors:** Daniel F. Chodosh, Francis E. Wdzieckowski, Julius Schainbaum, Charles E. Berkoff

**Affiliations:** Smith Kline & French Laboratories Inc. Preclinical Research & Development 1500 Spring Garden Street Philadelphia Pennsylvania 19101 USA


Journal of Automatic Chemistry, Volume 5, Number 2 (April–June 1983), pages 99–102

### Automated chemical synthesis. Part 2: Interfacing strategies

**Daniel F. Chodosh, Francis E. Wdzieckowski, Julius Schainbaum and Charles E. Berkoff**

*Smith Kline & French Laboratories, Inc., Preclinical Research & Development, 1500 Spring Garden Street, Philadelphia, Pennsylvania 19101, USA.*

Research focused largely on developing new, highly automated tools for the process development laboratory is carried out in the authors' laboratory. The ability to evaluate, characterize and optimize synthetic routes, both quickly and comprehensively, is especially important in the pharmaceutical process development laboratory, where typically in a complex synthetic sequence many reaction parameters must be examined. Furthermore, the rapid elucidation of process routes details and idiosyncrasies will allow the synthesis of new materials intended for extensive preclinical and clinical testing to proceed by 'final chemistry', where even slight changes in process conditions can affect trace impurity profiles. Increasing attention is being given to the opportunities generated by computers to enhance the productivity of these laboratories [1–2].

On the pilot and production scale, batch-reaction automation instrumentation and technology employing fully and semi-distributed control schemes are widely available [3–6]. On the laboratory scale, however, most researchers have pursued automation of continuous-flow techniques [7–9]. The automated chemical synthesis project described here entails designing and constructing a computer-controlled, bench-scale batch-type reactor, which is capable of self-directing experimentation and optimization [9–15]. Such a system would permit extensive, and often tedious, examination of chemical reactions in quite early stages of their development. Experimentation on the bench-scale in batch-type reactors will allow chemical reaction and process control data to be obtained, subsequently this will be directly applicable to the pilot and production scales. Further, by minimizing the size of the vessel, scarce developmental quantities of chemical intermediates can be conserved as automation technology is applied at the very earliest stages of process development programmes.

A prototype unit was constructed to permit a close evaluation of various automation parameters: reactor design, temperature control, reagent delivery, chemical analysis and computer interfacing. The unit consisted of a 100 ml glass (jacketed) vessel with ports for a condenser, stirrer bearing, temperature sensor, reagent inlet, reaction sampling and a drain. Reagent and solvent were introduced into the vessel by positive displacement pumps; temperature was controlled by mixing hot and cold fluids and circulating the mixture through the vessel jacket; samples were removed and diluted (using a Technicon AutoAnalyser pumping system) for subsequent on-line analysis by HPLC. A timeshare computing system (a Digital Equipment Corporation PDP11/34 RSTS) was used to calculate the various operational parameters (stoichiometry, temperature, reaction time) according to a simplex experimental design. The chemical analysis results were reported back to the RSTS resident program which then calculated new reaction parameters. For a detailed description of these systems the reader is referred to the preceding paper in this series [16].

Through this preliminary effort the project was shown to be suitably viable and a system-by-system evaluation and redesign was initiated. Instrument reliability was of particular concern; self-checking and fail-safe features also became fundamental considerations in the redesign efforts. To achieve greater reliability and more precise control the transfer of information between the computational system and the autosynthesis system was also scrutinized [17–19].

The prototype unit employs a timeshared computer to calculate reaction operating parameters. The RSTS resident program (in this case, a simplex optimization algorithm [10 and 14]) communicates via a single serial link (modem) to the laboratory (see figure 1). This timeshare environment does not support real-time functions, so it is not possible to place time-dependent instrument-control demands on the computing system. Once reaction parameters (time, temperature set-point, stoichiometry) are calculated they are passed to local digital controllers for execution. The flow of information is unidirectional, from RSTS to the controller. Each controller must be 'hardwired' to accept a parameter and independently actuate the requisite control function. For example, the reagent delivery controller accepts a serial string of ASCII characters corresponding to the number of required pump revolutions. The controller then activates the indicated pump until a hardwired feedback loop counts off the appropriate number of pump revolutions. The controller then deactivates the pump. The delivery rate of the pump, once manually set, remains fixed throughout the course of the experimental run. The computer system does not access information regarding the overall integrity of the synthesis apparatus (for example, reagent reservoir depletion, line fatigue or rupture, viscosity effects), or the completion status of the operation itself. In each case the instrument-control algorithm is fixed by the electronic design of the controller device. Each controller must be designed to communicate, albeit unidirectionally, with the computer in the identical manner (via serial modem), regardless of its control function or signal complement. While more sophisticated local controllers can be constructed,

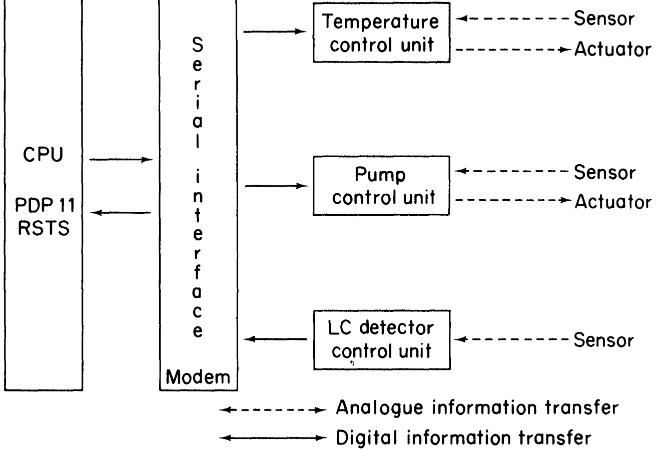

*Figure 1. Prototype unit: interface to timeshare computer system.*

this timeshare automation approach constrains the overall system by limiting the computer-synthesis systems to strictly non-time critical or dependent interactions and to a modest data collection.

By abandoning timesharing for real-time computing, significant advantages can be realized both in instrument design and performance. In recent years well-packaged microprocessor-based systems, complete with signal-acquisition interfaces (analogue-to-digital, digital-to-analogue, parallel I/O, etc.), have become available at reasonable cost. The capability to directly couple data signals between the synthesis apparatus and the computing system, through appropriate interfaces, totally redefines the automation problem (see figure 2).

Whereas, formerly, elements of decision-making were hard-wired in the various controllers (for example, P versus PI versus PID temperature control), decision-making can now be software resident. Changes to automation-control strategies can be implemented through facile software modification, as opposed to hardware modifications and/or redesign of the autosynthesis electronics. However, semi-distributed control elements are not necessarily avoided—indeed, certain aspects of the temperature control and fail-safe recovery automation in this system rely on semi-distributed control functionality. Rather, various autosynthesis systems can be selectively engineered through the range of non-distributed to semi-distributed—dictated only by the specific control problem at hand.

In this computing environment, substantially greater numbers of signals are available to the microprocessor for data collection and instrument control. With suitable mass-storage devices, the dedicated microcomputer is capable of extensive data collection which is inaccessible to the time-share environment. Thus data become available for a range of administrative and scientific tasks: for example, report writing, instrument activity logs, kinetic modelling studies, response surface mapping, cost analysis and impurity profiling. By choosing a real-time computing environment precise instrument control with auto-calibrating auto-correcting features is achievable. The experimentation can be self-directing, where the chemical analysis results of experiment $N$ are used to calculate the $N+1$ set of experimental conditions to be evaluated. Further, by real-time monitoring of the autosynthesis, electromechanical hardware system integrity can be assured allowing unattended operation; this provides better use of staff and cost savings.

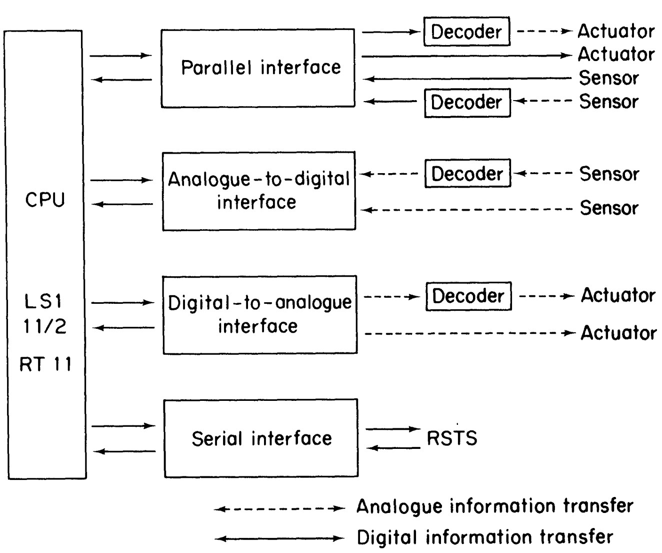

*Figure 2. Autosynthesis system: interface to dedicated computer system.*

Fail-safe recovery procedures were of fundamental concern throughout the re-evaluation. Assuming that the requisite automation hardware is present, verification of proper operation can be designed into the automation software. For example signals from flow monitors can be used to verify pump/syringe operations. Power failures and computer drop-out situations present additional challenges to the designer. To avoid a catastrophe in the laboratory the apparatus should default to a quiescent state until either automatic (i.e. computer) or manual recovery steps can be executed. A general-purpose interface was constructed to provide fail-safe protections.

A Digital Equipment Corporation MINC LSI 11/2 computer and associated interfaces were selected for the authors' application. The system configuration includes a dual hard disc storage system (RLO1), floppy discs (RXO2), a VT105 graphics CRT and a line-printer (shown in figure 3). The MINC interfaces provide excellent signal-handling capabilities: A/D (12 bit, successive approximation), D/A (12 bit, uni- and bipolar), digital input and output (16-bit registers) and a programmable clock. The real-time foreground/background operating system (RT11 V4 F/B) provides real-time computing capacity and access to a set of comprehensive scientific program libraries. Instrument-control and data-acquisition software is written in Fortran with real-time extensions (REAL-11/MNC). To utilize the digital input and output interfaces it was necessary to augment their functions. To protect the computer system from electrical anomalies (induction effects, sparking) these signal lines must be optically decoupled from the synthesis apparatus. The TTL signals available through the digital output interfaces (2 × 16 bits), while capable of interfacing to semiconductor logic, cannot directly couple to devices such as motors or solenoids. The opto-

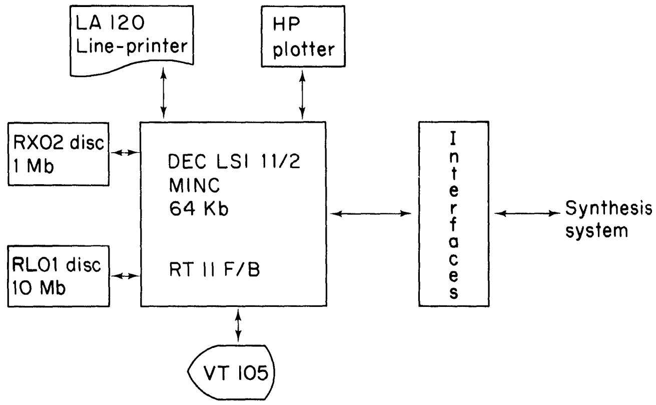

*Figure 3. Dedicated real-time computer configuration.*

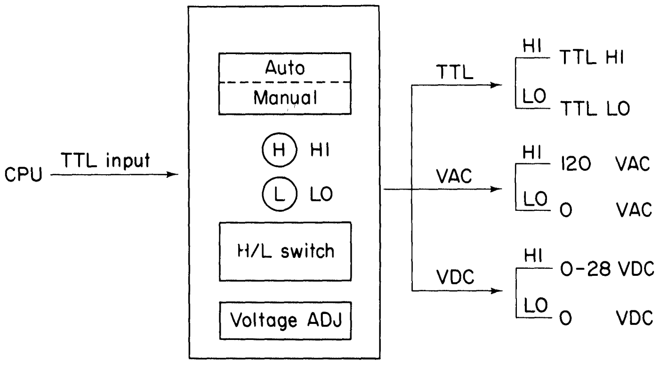

*Figure 4. Opto-isolator interface: single channel block diagram.*

isolator interface was designed to add new functions to these I/O interfaces and provide many of the default safeguards required.

As shown in figure 4, the opto-isolator interface accepts TTL signals from a 16-bit parallel output interface. Each signal line (i.e. bit) is electrically decoupled via an opto-isolator semi-conductor and propagated through the interface simultaneously, making available three signals for the synthesis apparatus: TTL, VAC and VDC. When the TTL input is low the outputs on the indicated channel are all low: TTL low, O VAC and O VDC. A TTL input high produces high output states: TTL high, 120 VAC and VDC. The d.c. voltage level in the high state is manually selected by adjusting a potentiometer on each channel. (The interface has a panel meter for voltage adjustments.) To operate a 24 VDC solenoid the user sets the indicated interface channel to 24 VDC; when the computer system generates a TTL high signal the output of the selected channel changes from 0 to 24 VDC driving the solenoid. The voltage range, 0–28 VDC, allows a wide variety of direct current devices to be interfaced easily and quickly. The individual channels have AUTO/MANUAL switches that select the channel input source. In the AUTO mode, the computer-generated signal drives the channel and determines the output state. In the MANUAL mode a toggle switch on each channel allows the user to select the output state; with this arrangement some of the channels can be driven by the computer while others are manually operated—this is useful during the testing and development of the automation software. To address the problem of computer drop-out, a 'sense line' couples the interface to a computer-generated TTL source. As long as the 'sense line' remains high the opto-isolator interface operates as described above. Should the 'sense line' go low, the AUTO/MANUAL switch is overridden and the outputs are set by the position of the toggle switches. This provides substantial default protections for the system. For example, the power-supplies for the heater systems are driven via the 120 VAC outputs of the interface. The operator leaves the appropriate toggle switches in the OFF position and selects the AUTO mode for normal computer operation. Should the computer drop out (i.e. the 'sense line' go low), the interface defaults to turn off the power-supplies which in turn prevents uncontrolled heating. In a similar fashion, default settings for the valves, motors and solenoids protects the synthesis apparatus from catastrophe. An additional 16-bit parallel output register is optically isolated and propagated (TTL output), as is a 16-bit parallel input register (signals from the apparatus to the computer system) making available additional 16 TTL input and 16 TTL output signals. Finally, a true-positive true-negative switch for logic polarity inversion is provided to assure general compatibility with other computer systems. A schematic diagram of one channel is shown as figure 5.

This computer environment and the interfaces described have sufficient signal- and data-handling capabilities for the automation experiment. With the addition of the opto-isolator interface, sufficient safeguards can be engineered into the laboratory apparatus to permit reliable unattended operation. The design of the synthesis apparatus itself, its interfaces to the computer and the automation software, will be described in future papers.

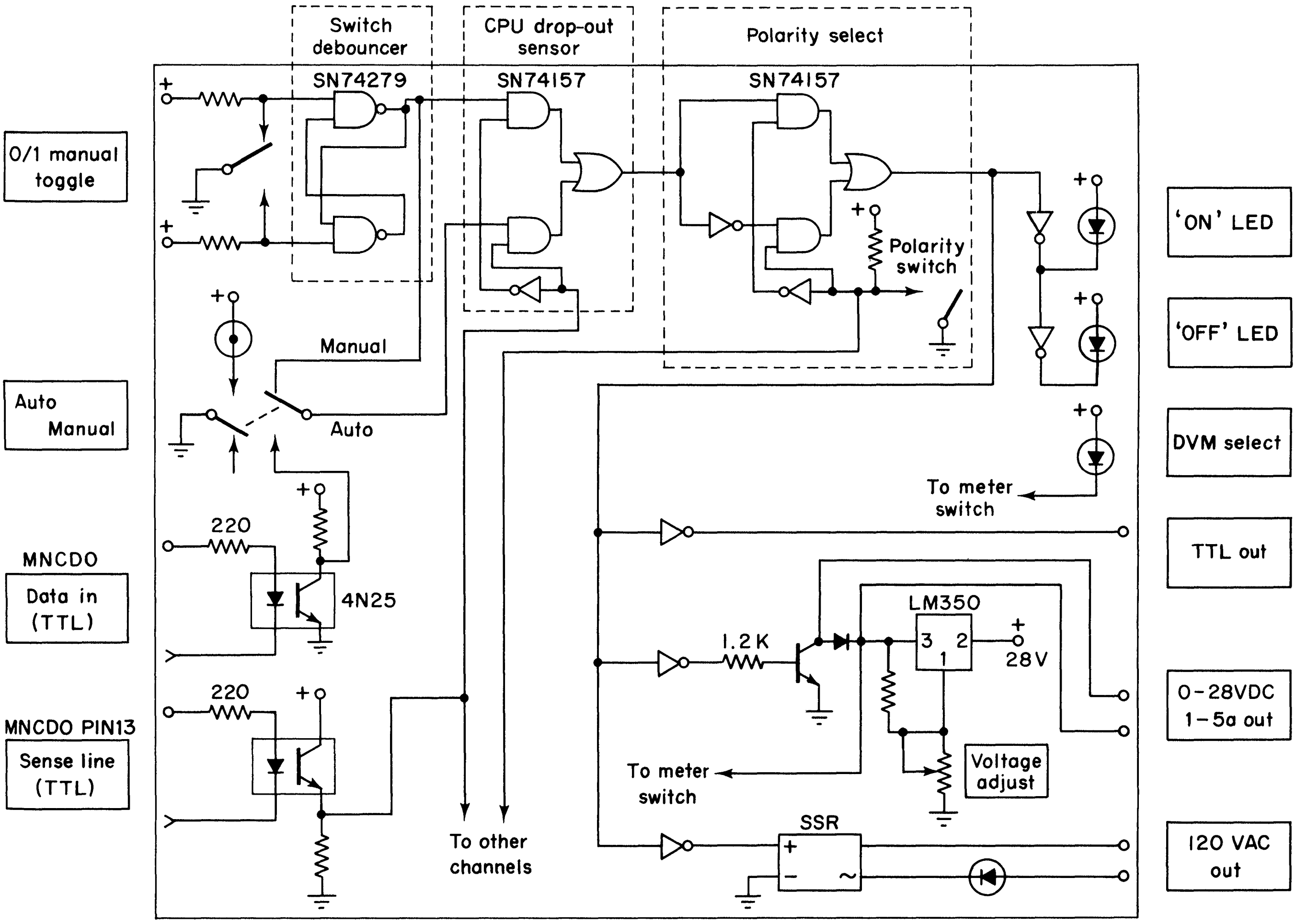

*Figure 5. Opto-isolator interface: single channel electrical schematic.*

### References

1. Walser, P. E. and Bartels, H. A., *American Laboratory* (1982), 113.
2. Frazer, J. W., *Accounts of Chemical Research*, **7** (1974), 141.
3. Brodmann, M. T. and Smith, C. L., *Chemical Engineering*, **83** (1976), 191.
4. Kennedy, J. P., *Chemical Engineering Progress*, **77** (1981), 33.
5. Merritt, R., *Inst. Cont. Sys.* (1981), 34.
6. Garton, R. D., *Chemical Engineering Progress*, **77**, (1981), 44.
7. Nagy, G., Feher, Z. and Pungor, E., *Analytica Chimica Acta*, **52** (1970), 47.
8. Spellman, R. A. and Quinn, J. B., AIChE Workshop in Industrial Process Control, Tampa, Florida (November 1974).
9. Frazer, J. W., Rigdon, L. P., Brand, H. R. and Pomernacki, C. L., *Analytical Chemistry*, **51** (1979), 1739.
10. Dean, W. K., Heald, K. J. and Deming, S. N., *Science*, **189** (1975), 805.
11. Deming, S. N., *American Laboratory* (1981), 42.
12. Box, G. E. P., *Biometrics*, **10** (1964), 16.
13. Frazer, J. W., Kray, A. M., Selig, W. and Lim, R., *Analytical Chemistry* **47** (1975), 869.
14. Deming, S. N. and Morgan, S. L., *Analytical Chemistry*, **45** (1973), 278A.
15. Watson, M. W. and Carr, P. W., *Analytical Chemistry*, **51** (1979), 1835.
16. Winicov, H., Schainbaum, J., Buckley, Jr., J. T., Longino, G., Hill, J. and Berkoff, C. E., *Analytica Chimica Acta*, **103** (1978), 469.
17. Chodosh, D. F., Winicov, H., Buckley, Jr., J. T. and Berkoff, C. E., Computers at the bench (Belgian Pharmaceutical Society International Conference on Computers in Pharmaceutical Research, Namur, Belgium, November 1979).
18. Chodosh, D. F., 1979 Fall D.E.C.U.S. Symposium, Workshop on Laboratory Automation, San Francisco (November 1978).
19. Chodosh, D. F., Buckley, Jr., J. T., Longino, G., Schainbaum, J., Wdzieczkowski, F. E., Winicov, H. and Berkoff, C. E., MINC interfacing (1981 D.E.C.U.S. Symposium, Los Angeles, December 1981).

### READER ENQUIRY SERVICE

Each advertisement and each editorial item in the Product News section in *Journal of Automatic Chemistry* carries a number which corresponds with a number on the Reader Enquiry Card bound into every issue.

If there are any items on which you would like further information, circle the relevant number on the card, indicate the volume and issue number, fill in your name and full address, and return the card to Taylor & Francis Ltd. Your card will then be forwarded to the company/ies concerned for action.

*Through using the Reader Enquiry Service, you can obtain information on a variety of products through one simple operation.*

### GAS RESEARCH CONFERENCE

More than 100 papers on a variety of topics involving gas research will be presented and discussed during the 1983 International Gas Research Conference which will take place at the London Hilton Hotel from 13–16 June. This is the first time that this conference has been held outside the United States. Sponsors of the conference are the Gas Research Institute, the International Gas Union, the American Gas Association and the United States Department of Energy. The British Gas Corporation and the Institution of Gas Engineers will act as hosts.

The five broad categories into which papers are divided cover: distribution and transmission; substitute natural gas; domestic and commercial utilization; industrial utilization; and thermophysical properties and processes. In addition there will be an opening address by the Chairman of the British Gas Corporation, Sir Denis Rooke; and keynote speeches by Christoph Brecht (FR of Germany), President of the IGU; George H. Lawrence (USA), President of the AGA; and Henry R. Linden (USA), President of the GRI. Other speakers will include Ulf Lantzke (France), Director General of the International Energy Agency; Sir Hermann Bondi (UK), Chairman of the Environment Research Council; and John Kean (USA), Vice-President of the IGU.

English and French will be the official languages of the conference, with simultaneous translation facilities for all technical papers.

Registration fee for delegates is £290; an accompanying persons' programme is available for £80.

*Full details of the technical programme, tours, hotel information and registration forms are available from: International Gas Research Conference, c/o Conference Services Ltd, 3–5 Bute Street, London SW7 3EY. Tel.: 01 584 4226.*

### EDITORIAL NOTE

The Editor is pleased to receive new books on chemical automation and mechanization. Review copies should be sent to:

*Dr Peter Stockwell, Plasma-Therm Ltd, Unit 3, 2/3 Kangley Bridge Road, Lower Sydenham, London SE26 5AR.*

